# Depressive symptoms related to low fractional anisotropy of white matter underlying the right ventral anterior cingulate in older adults with atherosclerotic vascular disease

**DOI:** 10.3389/fnhum.2015.00408

**Published:** 2015-07-15

**Authors:** Kelly R. Bijanki, Joy T. Matsui, Helen S. Mayberg, Vincent A. Magnotta, Stephan Arndt, Hans J. Johnson, Peg Nopoulos, Sergio Paradiso, Laurie M. McCormick, Jess G. Fiedorowicz, Eric A. Epping, David J. Moser

**Affiliations:** ^1^Department of Psychiatry, The University of Iowa Carver College of Medicine, Iowa City, IAUSA; ^2^Department of Psychiatry, Emory University School of Medicine, Atlanta, GAUSA; ^3^Department of Biomedical Engineering, University of Iowa College of Engineering, Iowa City, IAUSA; ^4^Department of Neurology, Emory University School of Medicine, Atlanta, GAUSA; ^5^Department of Radiology, The University of Iowa Carver College of Medicine, Iowa City, IAUSA; ^6^Department of Biostatistics, University of Iowa College of Public Health, Iowa City, IAUSA; ^7^Núcleo UDP-Fundación INECO para las Neurociencias, Facultad de Psicología, Universidad Diego Portales, SantiagoChile; ^8^Department of Epidemiology, University of Iowa College of Public Health, Iowa City, IAUSA

**Keywords:** diffusion tensor imaging, atherosclerosis, subgenual cingulate, subcallosal cingulate, depression, aging, fractional anisotropy, mean diffusivity

## Abstract

We sought to characterize the relationship between integrity of the white matter underlying the ventral anterior cingulate (vAC) and depressive symptoms in older adults with atherosclerotic vascular disease (AVD), a condition associated with preferential degeneration of the white matter. The vAC was defined as including white matter underlying ventral Brodmann Area 24 and Brodmann Area 25, corresponding with the “subcallosal” and “subgenual” cingulate respectively. This region of interest was chosen based on the preponderance of evidence that the white matter in the region plays a critical role in the manifestation of depressive symptoms. Participants had current unequivocal diagnoses of AVD and were between 55 and 90 years-old. Fractional anisotropy (FA) was used as an index of white matter integrity and organization. Whole-brain mean diffusivity (MD) was used as an index of global white matter lesion burden. Depressive symptoms were measured using the Symptom Checklist-90-Revised (SCL-90-R) Depression Scale. Depressive symptoms were significantly related to low FA in the right vAC (*r* = -0.356, df = 30, *p* = 0.045) but not the left vAC (*r* = 0.024, df = 30, *p* = 0.896) after controlling for total brain MD (a statistical control for global white matter lesion burden). Further, depressive symptoms were significantly related to low FA in the right vAC (*r* = -0.361, df = 31, *p* = 0.039), but not the left vAC (*r* = 0.259, df = 31, *p* = 0.145) when controlled for the contralateral vAC FA. The correlation coefficients for this follow-up analysis were found to be significantly different between left and right vAC (*Z* = 2.310, *p* = 0.021). Poor white matter health in the vAC may be a biological mechanism for depressive symptoms in older adults with vascular disease. Further studies may corroborate that the right vAC plays a unique role in depressive symptom manifestation in cases where the white matter is preferentially affected, as is the case in AVD. This could lead to future targeting of the region for somatic antidepressant treatment, as well as the development of a precise approach for patients with white matter damage, which could produce significant improvement in quality of life, medical morbidity, and mortality.

## Introduction

The vAC has been established as a critical hub of the putative depression network, given evidence of its anatomical connectivity with the regions in the limbic-cortical depression circuit (Johansen-Berg et al., 2008; Rudebeck et al., 2014). The vAC includes ventral Brodmann Area 24 and Brodmann Area 25, termed subcallosal and subgenual cingulate respectively (Bijanki et al., 2014). Converging evidence from an array of neuroimaging modalities and participant groups has implicated the vAC in depressive symptom manifestation (Mayberg, 2009). For example, elevated blood flow in the vAC gray matter is noted in antidepressant treatment non-responders (Greicius et al., 2007), currently depressed (Dougherty et al., 2003; Mayberg et al., 2005; Kennedy et al., 2007), and transiently sad healthy normal participants (Mayberg et al., 1999; Zald et al., 2002). Changes in blood flow or glucose metabolism in the vAC gray matter are also noted in response to various interventions, such as antidepressant medication (Mayberg et al., 1999; Kennedy et al., 2007), placebo treatments (Mayberg et al., 2002), electro-convulsive therapy (Nobler et al., 2001), and cognitive-behavioral therapy (Kennedy et al., 2007). Further, cellular abnormalities have been reported in the white matter underlying the vAC in postmortem depressed patient samples (Drevets et al., 1997; Ongur et al., 1998; Rajkowska and Miguel-Hidalgo, 2007), and the critical white matter fiber tracks associated with symptom improvement in treatment-refractory depression have been characterized as emanating from the white matter underlying the vAC (Riva Posse et al., 2014). Based on the extensive past literature implicating the vAC in depressive phenomenology, the current study has taken a hypothesis-driven approach. We sought to examine the effects of vAC white matter health on depressive symptoms, by examining patients with ischemic damage to the white matter without primary diagnoses of depression. The current study has utilized a dimensional symptom approach with the goal of avoiding common confounders such as physician diagnostic patterns, and patients’ access to psychiatric care.

This study examined a sample of patients with AVD, a common condition among older adults, and one with previously demonstrated adverse effects on white matter in the context of relatively spared gray matter (Bijanki et al., 2013a). In AVD, the blood vessels develop atherosclerotic plaques, which protrude into the vessel lumen preventing adequate blood flow and leading to hypoperfusion of the target tissues (Mitchell and Schoen, 2010). If the level of blood perfusion cannot provide the necessary oxygen and glucose to support cellular metabolism, ischemic damage develops in the tissue. In atherosclerosis, the white matter tends to be preferentially affected by ischemic damage (Pantoni et al., 1996), likely owing to greater vulnerability of its vasculature to luminal narrowing. By comparison, the cortex has a built-in defense mechanism from the Circle of Willis, which provides a back-up perfusion structure in the case that one of the contributing arteries becomes occluded (Alpers et al., 1959).

The link between vascular disease and depression has been previously articulated in the vascular depression hypothesis, which contends that cerebrovascular disease may precipitate or perpetuate depressive symptoms in patients with significant vascular disease (Alexopoulos et al., 1997; Krishnan et al., 1997). White matter hyperintensities (areas of presumed ischemia indicated by bright spots on fluid-attenuated inversion recovery (FLAIR) magnetic resonance neuroimaging) are the most common neuroradiological finding in participants with vascular disease (Taylor et al., 2013). The recent longitudinal multicenter pan-European study, “Leukoaraiosis and Disability in the elderly (LADIS),” has shown a relationship between the progression of white matter changes (identified as white matter hyperintensities on FLAIR images) and late life depression symptoms (Firbank et al., 2012). More recently, this work was extended by Khalaf et al. (2015), who showed significant global white matter hyperintensity accumulation over a 12-weeks antidepressant treatment course in patients with late life depression, which was specific to treatment non-responders. While effects of global lesion burden have been previously demonstrated in vascular disease, the specific contribution of the vAC has never before been examined.

Advances in the field of neuroimaging allow us to take a more sensitive look at the organization, health, and implicit connectivity of the white matter in the brain. Diffusion tensor imaging (DTI) is an important method to further study the white matter, which works by applying a series of diffusion-sensitizing gradients to the brain and measuring the extent to which water molecules are able to diffuse in the direction of the gradient. The most commonly reported measure in DTI studies is FA, which reflects to the degree to which diffusion is directionally constrained by white matter fibers (Basser and Pierpaoli, 1996). Higher FA is taken to indicate healthier, more organized white matter tracts (Assaf and Pasternak, 2008). FA is considered a non-specific marker of tissue pathophysiology; abnormalities (decreases) in FA have been reported from a wide variety histological sources, including demyelination, edema, inflammation, and gliosis (Assaf and Pasternak, 2008). By comparison, MD, sometimes termed simply “diffusivity,” reflects the degree of diffusion in a given voxel, regardless of the direction. Higher MD is interpreted to most strongly indicate ischemic white matter damage, as demonstrated by its high degree of correlation with measures of white matter lesion volumes manually identified from FLAIR images (Shimony et al., 2009), which are commonly observed in vascular disease with known association to depressive symptoms (Taylor et al., 2003).

The current study examined the relationship between white matter integrity in the vAC and depressive symptoms in patients with AVD. We expected in the current sample of patients with AVD that there would be a significant relationship between poor white matter health in the vAC and elevations in depressive symptoms, based on previous findings implicating the region as a hub, and based on the vascular depression hypothesis suggesting vascular disease leads to ischemic white matter damage which is associated with depressive symptoms.

## Materials and Methods

### Participants

As a part of the parent study, “Aging, Vascular Disease, and Cognition,” (grant number R01AG030414-01A2 from the National Institute on Aging), older adult participants aged 55–90 were recruited through the University of Iowa Heart and Vascular Care clinic, and healthy comparison (HC) participants were recruited through newspaper and magazine advertisements. All AVD participants had unequivocal clinical diagnoses of AVD and a history of one or more of the following: angina pectoris, myocardial infarction, percutaneous transluminal coronary angioplasty, placement of coronary artery stent, or peripheral vascular disease (claudication). Exclusion criteria included more invasive cardiovascular procedures, stroke, epilepsy, dementia, head injury, and focal neurological signs as described elsewhere (Moser et al., 2007). HC participants were required to meet none of the AVD inclusion or exclusion criteria. All procedures were conducted in accordance with the ethical standards of the institutional review board of the University of Iowa and the Helsinki Declaration of 1975. Informed consent was obtained from all patients prior to initiating any study procedures. Participants then provided demographic data and received a complete history and physical assessment.

Of the parent study AVD participants, 36 took part in neuroimaging. Unfortunately, one scan was unsuitable for DTI analyses because of abnormal warping in the posterior lobes. Therefore, 35 AVD patients participated in the current study with a mean age of 67.2 (7.4 SD), mean education of 14.8 years (3.3 SD), including 14 women and 21 men who were predominantly right-handed (29 right-handed, 5 left-handed, 1 ambidextrous). 25.7% of participants were treated with antidepressants, and the average score on the SCL-90-R Depression Scale was 53.1 (9.4 SD). No participants in the current study used antipsychotic or antiepileptic medications.

### Procedures

The SCL-90-R (Derogatis and Melisaratos, 1983) was used by the parent study to measure depressive symptoms, represented as T-scores and adjusted to separate norms for men and women, where higher scores indicate more severe symptoms. The SCL-90-R Depression Scale has shown strong correlation with the Beck Depression Inventory (Beck et al., 1988), suggesting convergent validity (Steer et al., 1994). Previous analysis by our group has shown that scores on the SCL-90-R are suitably sensitive to show relationships with brain structural measures (Bijanki et al., 2013b). In the parent study of 111 AVD and 51 HC participants, the AVD participants had significantly elevated SCL-90-R depression scores compared with the HC participants (AVD mean 55.18, HC mean 50.67, *t* = 2.466, df = 160, *p* = 0.015), supporting the premise that AVD participants experience elevated levels of depressive symptoms. The full range of SCL-90-R scores in the current neuroimaging sample is demonstrated in Supplementary Figure S1. The data cover a broad range, including participants as far as 2.5 SD above the mean and approximately 1.5 SD below the mean. In the current sample, there were six participants with scores more than 1.5 SD above the mean, which may be consistent with clinically significant depressive illness in spite of a high rate of antidepressant medication use.

Participants underwent anatomical and DTI using a Siemens Avanto 1.5T magnetic resonance imaging scanner (Erlangen, Germany). DTI data were gathered on 56 slices using an interleaved slice acquisition, slice thickness of 2.5 mm, gap between slices of 0 mm, in a 256 mm × 256 mm field of view. The acquisition matrix was 128 × 128, the reconstruction matrix was 128 × 128, using 12 directions and a b-value of 1000 s/mm^2^. TR/TE = 9500/94 ms, flip angle was 90°, pixel bandwidth was 1347 Hz, and the imaging frequency was 63.623624 MHz. The anatomical images collected included a 3D T1 weighted scan, 2D T2 weighted scan, and a 2D FLAIR scan. The 3D T1 weighted images were collected in the coronal plane using a FLASH sequence (TE = 4.7 ms, TR = 25 ms, flip angle = 30°, field of view = 260 mm × 260 mm × 192 mm, matrix = 256 × 256 × 128, averages = 2, bandwidth = 130 Hz/pixel). The T2 weighted images were acquired in the coronal plane using a fast spin-echo sequence (TE = 95 ms, TR = 9750 ms, field of view = 260 mm × 260mm, matrix = 256 × 256, slice thickness/gap = 3.0/0.0 mm, averages = 2, bandwidth = 130 Hz/pixel, echo train length = 20). The FLAIR images were collected in the axial plane (TE = 110 ms, TR = 9600 ms, TI = 2500 ms, field of view = 260 mm × 260 mm, matrix = 256 × 256, slice thickness/gap = 3.0/0.0 mm, averages = 2, bandwidth = 130 Hz/pixel, echo train length = 15).

Automated processing of the anatomical images was performed using BRAINS2 software (Magnotta et al., 2002). The BRAINS2 software includes automated AC–PC alignment, image alignment, image intensity standardization, tissue classification, and brain extraction. The BRAINS2 method for white matter segmentation has shown reliability with manual raters and adequately addresses concerns of partial volume contamination (Harris et al., 1999). Only the T1 and T2 weighted images were considered in the tissue classification and thus the white matter lesions were initially classified as gray matter. To correct this artifact, the FLAIR and tissue classified images were consulted and a human rater (KB) manually defined the white matter lesions. It was determined that all white matter tissue should be included in the DTI analysis regardless of whether it was categorized as “lesioned” or “unlesioned” based on the FLAIR image, so the manually segmented lesion volumes were added into the white matter masks used to define the diffusion-weighted data reported in the current study.

The diffusion-weighted images were co-registered to the B0 image, and diffusion-weighted data underwent automated quality control using the DTIPrep package (NITRC), as well as subsequent visual inspection for quality control to check for artifacts such as head motion, table vibration, RF noise, and susceptibility artifacts. Motion correction and diffusion tensor decomposition were performed and diffusion tensors and scalars were calculated from 12 diffusion-sensitizing gradients, using GTRACT software (Cheng et al., 2006). The B0 image was then registered with the anatomical image, and the resulting transform was applied to the FA and MD images.

The vAC was segmented by applying a well-validated white matter atlas (Mori and Van Zijl, 2007) to each participant’s neuroimaging data, which had been revised to include a vAC parcellation. There are no clear anatomical boundaries the anterior “subgenual” (ventral Brodmann Area 24) and posterior “subcallosal” (Brodmann Area 25) subregions, and there is extensive evidence implicating both subregions in the manifestation of depressive symptoms across a variety of neuroimaging methods (Ongur et al., 1998; Rajkowska and Miguel-Hidalgo, 2007; Johansen-Berg et al., 2008), so the current study has sampled the vAC widely using the standardized atlas.

**FIGURE 1:**
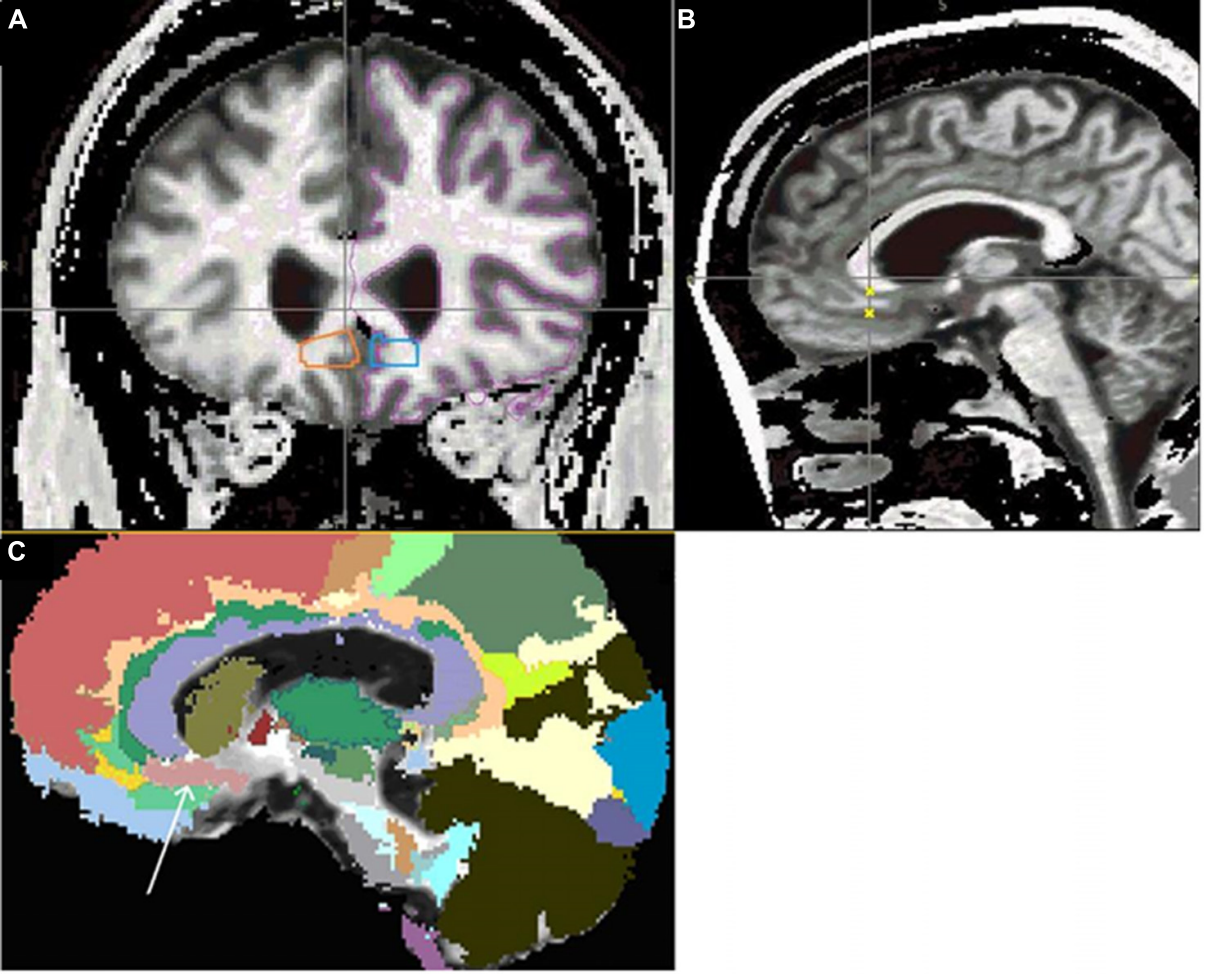
**Segmentation of vAC via manual traces integrated into automated atlas. (A)** Demonstrates manual vAC traces in orange and blue. The magenta outline demonstrates the extent of the white matter mask. **(B)** Shows small yellow x marks on the vertical crosshair to indicate where the orange trace intersects with the sagittal plane shown. ROIs are inclusive of gray matter which is excluded via the application of the white matter mask (shown in purple on left hemisphere of coronal image) prior to FA calculation. **(C)** Shows sagittal slice with Mori atlas label map overlay, showing the extent of the masked vAC region of interest (peach area below the violet genu of the corpus callosum), indicated by the white arrow.

The vAC region of interest was defined based on a previously published method as follows: the anterior boundary was the first slice after the corpus callosum joined the two hemispheres, the posterior boundary was the end of the paraterminal gyrus, the superior boundary was defined by the corpus callosum, and the inferior boundary was the inferior limit of the cingulate sulcus, the lateral boundary was the most inferior tip of the lateral ventricle, and the medial boundary was the ending of the white matter (McCormick et al., 2006). These manual traces were reviewed by the neuroimaging team (JM, VM, HJ, PN, LM, and DM) and integrated into the white matter atlas. Next, the revised atlas was registered to the anatomical images and scalar maps, using a B-Spline registration with a 12×12×12 grid size and a maximum displacement of 8 mm for any of the grid control points, and FA values were generated for vAC regions bilaterally. **Figure 1** shows the coverage of the vAC achieved by this technique.

### Analyses

#### Region of Interest Analysis

Bivariate correlations were used to examine the relationships between demographic variables (age, sex, education, and antidepressant use) and FA measures in the vAC white matter to help determine the appropriate control structure for subsequent Pearson partial correlations between FA and depression scores on the SCL-90-R. To characterize white matter lesion burden, we calculated global MD scores. In the current sample, whole-brain MD values strongly correlate with manually traced white matter lesion volumes in the whole brain (*r* = 0.528, df = 35, *p* = 0.001), suggesting convergent validity. Then, FA in the vAC was correlated with SCL-90-R depression scores, with statistical control for age and whole-brain MD in the white matter (a proxy for global white matter lesion burden). Tests of normality of the FA and SCL-90-R data indicated that the use of parametric analyses was appropriate in the current sample.

Next, we performed an analysis with the goal of further characterizing the hemispheric specificity of the relationship between white matter health and depressive symptom variability. This analysis controlled for FA in the contralateral vAC region in addition to the previously included demographic covariate (age). Although studies do not frequently use contralateral controls, the contralateral vAC white matter was chosen as the control region because it included the same volume of white matter with similar anatomical properties (such as branching of white matter tracts) as the region of interest, and it allowed for a more intensive examination of the hemispheric specificity of the findings. In addition to the statistically significant relationship identified in this follow-up analysis, we examined whether there were differences in mean FA between the right and left hemispheres in the vAC, using paired-samples *t*-test, and we examined whether there was a statistically significant difference in correlation coefficients between the right and left hemisphere in the relationship of FA with SCL-90-R Depression Scale scores, using an extension of the Fisher Z transformation to test the significance of differences between two correlated correlation coefficients (Meng et al., 1992).

## Results

Fractional anisotropy values in the vAC were examined for significant covariates, including age, education, sex, handedness, and antidepressant use. Consistent with previous literature (Pfefferbaum et al., 2005), the correlation between FA in the vAC white matter and age was found to be significant (*r* = -0.48, df = 33, *p* = 0.003). Non-significant correlations were found with education (*r* = 0.11, df = 33, *p* = 0.52). There were no significant differences in mean FA in the vAC as a function of sex (*t* = -0.55, df = 33, *p* = 0.59), right/left handedness (*t* = 1.07, df = 32, *p* = 0.30), or antidepressant use (*t* = -0.837, df = 33, *p* = 0.409). SCL-90-R Depression T-scores are routinely normed for sex, but they showed no significant correlations with age (*r* = -0.14, df = 33, *p* = 0.42) or education (*r* = -0.06, df = 33, *p* = 0.72). Therefore, partial correlations between FA and depression T-scores were statistically controlled for only age (among the possible demographic covariates).

Partial correlation between vAC FA and depression T-scores in participants with AVD showed a significant relationship (*r* = -0.356, df = 30, *p* = 0.045) in the right hemisphere when statistically controlled for total cerebral white matter diffusivity (MD) and age (**Table 1**). The analogous relationship between left vAC FA and depressive symptoms was not statistically significant (*r* = 0.024, df = 30, *p* = 0.896).

**Table 1:** Pearson partial correlation between FA in the vAC white matter and depression score, controlled for age and total white matter MD.

	df	*r*	*p*-value
Left vAC FA	30	0.024	0.896
Right vAC FA	30	-0.356	0.045*

Depression score reflects the sex-normed T-score for depressive symptoms on the SCL-90-R. FA, fractional anisotropy; vAC, ventral anterior cingulate; df, degrees of freedom; *, statistically significant.

**FIGURE 2:**
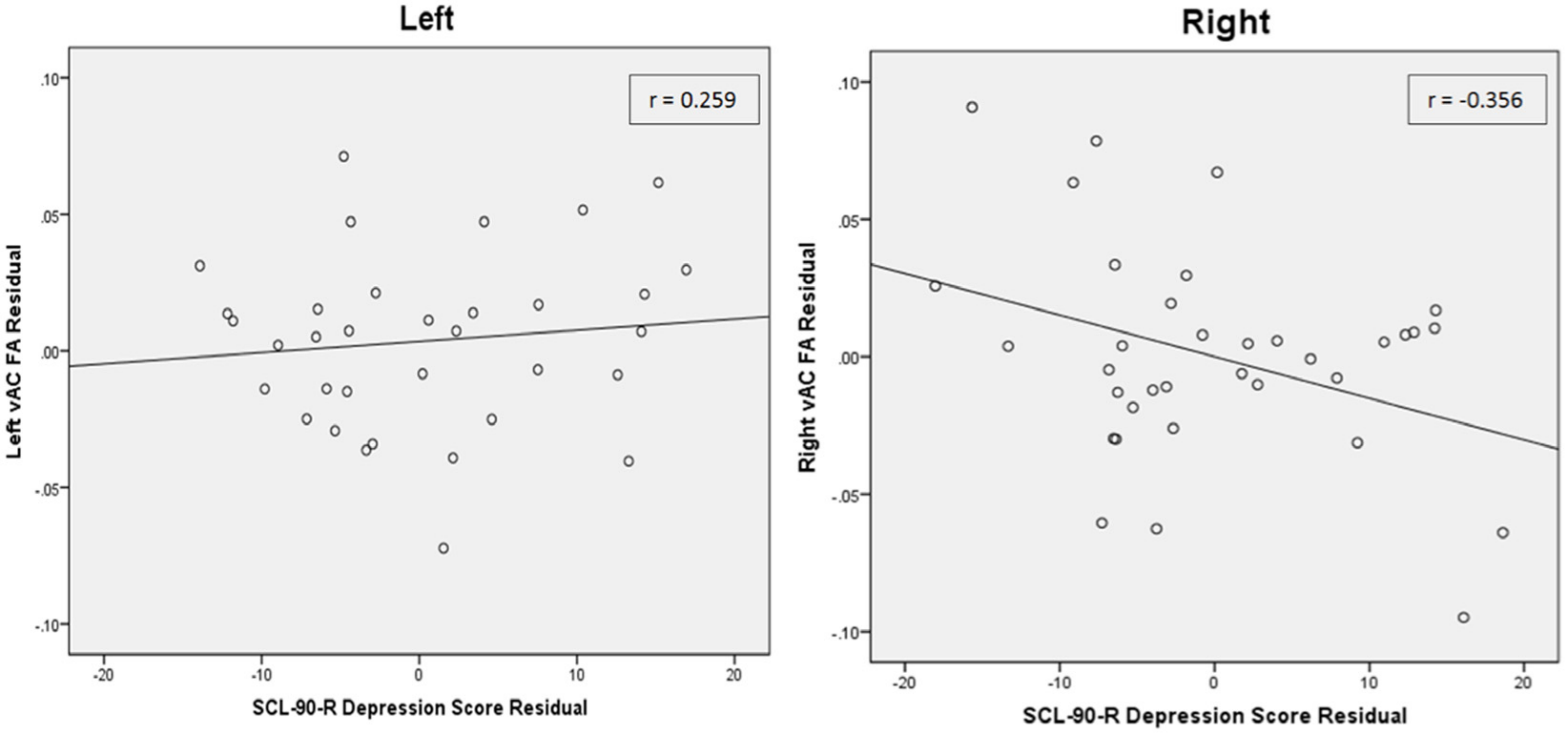
**Partial correlations between depressive scores and vAC FA values in the right and left. (Left)** Demonstrates the partial correlation between depressive score and left vAC FA, residualized against age and contralateral vAC FA. **(Right)** Demonstrates the significant partial correlation between depressive score and right vAC FA, residualized against age and contralateral vAC FA. Inset legends indicate partial correlation coefficients, and lines indicate linear regression fit line using the least squares method. To visualize these partial correlations, the independent and dependent variables were regressed onto the covariates using a linear regression. The resulting unstandardized residual variables were computed for each participant and saved, and then Pearson correlations were run on the residual variables. Residuals refer to variance left over in the correlated variables beyond variance shared with covariates.

Partial correlation between vAC FA and depression T-scores in participants with AVD also showed a significant relationship (*r* = -0.361, df = 31, *p* = 0.039) in the right hemisphere when statistically controlled for FA in the contralateral vAC region and age (**Table 2**; **Figure 2**). The analogous relationship between left vAC FA and depressive symptoms was non-significant (*r* = 0.259, df = 31, *p* = 0.145). Controlling for other contralateral white matter structures yielded similar correlations which did not reach the level of statistical significance.

**Table 2:** Pearson partial correlations between FA in the vAC white matter and depression score, controlled for age and FA in the contralateral ventral anterior cingulate region.

	df	*r*	*p*-value
Left vAC FA	31	0.259	0.145
Right vAC FA	31	-0.361	0.039*

Depression score reflects the sex-normed T-score for depressive symptoms on the SCL-90-R. FA, fractional anisotropy; vAC, ventral anterior cingulate; df, degrees of freedom; *, statistically significant.

A comparison of mean FA in the left and right vAC white matter showed no significant differences (*n* = 35, *t* = 0.985, *p* = 0.332) using a paired-samples *t*-test. Comparison of the correlation coefficients showed that the relationship of right vAC FA with SCL-90 depression was significantly different than the relationship of left vAC FA with SCL-90 depression (*Z* = 2.310, *p* = 0.021; **Figure 2**).

## Discussion

A significant association between FA in vAC white matter and depressive symptoms was observed in participants with AVD. In the current study, we examined FA in the vAC with statistical control for white matter diffusivity in the whole brain (MD). In this context, MD is used as a proxy measure of global white matter lesion burden, which is a common neuroradiological finding in patients with vascular disease (Taylor et al., 2003). Without controlling for global white matter lesion burden, it would be impossible to disentangle whether the finding in our region of interest was driven by local effects or by global white matter damage (for example, if AVD patients with poorer white matter health in the whole brain tended to have higher depressive symptom endorsement, an effect described by Shimony et al. (2009) in a different patient group). The relationship between vAC FA and depression was found to be statistically significant above and beyond the previously described relationship between depressive symptoms and whole-brain MD (Shimony et al., 2009), suggesting a critical role for this brain region in particular (**Table 1**). Further, the current findings suggest there may be a non-ischemic pathophysiological abnormality in the region, given that FA is sensitive to many non-ischemic sources of white matter disturbance (Assaf and Pasternak, 2008) and the finding has been statistically controlled for ischemic white matter lesions (MD).

We then carried out further analyses to examine the hemispheric specificity of our finding. In the second analysis, we examined the correlation between FA in the right vAC white matter and depression scores, after statistical control for FA in the contralateral vAC region. This control region was selected because it included the same volume of white matter with similar anatomical properties (such as branching of white matter tracts) as the region of interest, and it allowed for an intensive examination of the hemispheric specificity of the findings (Iwabuchi et al., 2011). This analysis revealed a significant correlation between depressive symptoms and FA in the right vAC white matter, as well as a non-significant correlation in the left vAC in the opposite direction (**Table 2**; **Figure 2**). By using an extension of the Fisher Z transformation, we found that the correlation coefficients for the left and right vAC regions were significantly different (Meng et al., 1992). Finally, we asked whether the laterality of our finding could be explained by simple differences in white matter integrity or organization in the left vs. right vAC in our sample. We compared mean FA values between the left and right vAC using paired-samples *t*-tests, and found that there was no difference between the two, suggesting a difference in function (rather than structure) between the hemispheres. Laterality studies on the relationship between white matter health and depression are mixed. There is significant historical evidence that stroke damage to the left hemisphere gray matter is associated with elevated depressive symptoms (Robinson and Price, 1982), but there is growing evidence that poor white matter health on the right side of the brain is associated with depressive symptoms in the elderly (Taylor et al., 2004; Bae et al., 2006).

The study of white matter health in AVD is complicated by the presence of white matter lesions, which have significant effects on one of the most common methods for whole-brain DTI analysis – tract-based spatial statistics or TBSS. In TBSS, white matter lesions have been found to produce unpredictable results on the skeletonized FA image used to define the common tracts for voxel-wise cross-subject statistical analysis (Cercignani, 2011). In the absence of appropriate and statistically powerful whole-brain DTI analyses for use in patients with AVD, as well as based on the preponderance of evidence suggesting a hypothesis-driven examination of the vAC, the current study sought to focus on thoroughly characterizing the relationship between white matter health in one specific region of interest (vAC) and depressive symptoms. By comparison to whole-brain exploratory analyses, the statistical power of examining only an *a priori* defined region of interest via the application of a white matter atlas is much greater. The ROI analysis in the current study also allowed for statistical control for whole-brain MD (lesion burden), thereby addressing the issue of regional specificity that would otherwise need to be addressed by a whole-brain analysis.

A variety of neurological and psychiatric illnesses are proposed to arise from the disconnection or hyperconnection of functionally coordinated cortical units (Geschwind, 1965; Catani and ffytche, 2005). Based on the findings in the present study and others (Grool et al., 2013), we propose that the elevation of depressive symptoms in participants with vascular disease is related to the accumulation of white matter damage in the critical limbic cortical white matter pathways. Our findings are in agreement with the finding of previous DTI studies on elderly patients with depressive symptoms (Taylor et al., 2004), including the right-lateralized FA finding (Bae et al., 2006). The findings of the current study are especially noteworthy, given that significant correlation coefficients were detected, ranging between *r* = 0.356 and *r* = 0.361. These correlations are generally consistent with the literature relating brain structure and mental functions. For instance, a study examining DTI in a group of 75 depressed and 23 control participants reported significant findings with correlations between *r* = 0.26 and *r* = 0.32 (Shimony et al., 2009).

We speculate the current findings are specific to white matter pathology, and possibly related to a type of non-ischemic change in white matter that occurs in AVD. The current study made use of an AVD sample because of the interesting phenomenon that brain changes are constrained to the white matter due to the anatomy of the supporting vasculature. With changes constrained to the white matter, we are able to more clearly examine the role of the white matter in depressive symptom manifestation, in the absence of gray matter lesions often observed in other types of vascular disease. The clinical relevance of studying AVD is also clear, in that AVD is a very common condition and takes place at a relatively early stage where aggressive treatment could alter the course of disease.

The current study is limited by basic experimental issues: a moderately small sample, only one measure of depressive symptoms, and the use of 1.5 Tesla MRI imaging with only 12 diffusion-sensitizing gradient directions. Further studies are needed to replicate the observed relationship and extend the current findings to include tractography analysis from the vAC to mood-related brain regions in the limbic and cortical systems. Furthermore, future studies should aim to elucidate the extent to which current findings are specific to AVD as opposed to other forms of vascular disease, as well as examining whether the current findings are specific to vascular disease as opposed to patients with depressive symptoms without vascular disease. Additional study is needed to explore whether the relationship between white matter health in the vAC and depressive symptoms can be demonstrated in aging healthy control participants, who are known to undergo a base rate of subtle white matter lesion accumulation. In addition, a prospective longitudinal analysis could examine the causality of the relationship between white matter damage and depressive symptoms, which remains unclear. While there is a convergence of evidence that depressive symptoms can arise from white matter changes in the brain caused by hypoperfusion in vascular disease, there is significant evidence suggesting that depression can cause cardiovascular abnormalities as well (see Musselman et al., 1998 for a review).

The current study has shown that in older adults with vascular disease, more severe depressive symptoms are related to lower anisotropy (a putative indicator of poor white matter health) in the white matter underlying the right vAC. These findings suggest an important role for the health of white matter at the vAC hub for mood regulation. These findings, with appropriate replication and expansion, may lead to the development of better treatment approaches for older adults with vascular disease, with the added goal of identifying those at greatest risk for developing depressive symptoms. Based on recent findings by Khalaf et al. (2015), we posit that aggressive treatment of vascular disease may be an important mitigator of white matter degeneration, which is related to depression as well as treatment non-response in older adults. Further, somatic interventions for depression may be more effective when targeted to the right hemisphere in older adults with vascular disease. We hope that greater understanding of the neural substrates of depressive symptoms will help inform treatment strategies for patients with vascular disease.

## Author Contributions

KB, DM, HM, PN, VM, SP, SA, HJ, and LM contributed to the design of the work. KB, JM, VM, and HJ contributed to the acquisition and work-up of the neuroimaging data. KB, DM, HM, SP, LM, SA, PN, VM, JF, and EE contributed to the data interpretation and drafting the work.

## Conflict of Interest Statement

Drs Kelly R. Bijanki, Joy T. Matsui, Vincent A. Magnotta, Stephan Arndt, Hans J. Johnson, Peg Nopoulos, Sergio Paradiso, Laurie M. McCormick, Jess G. Fiedorowicz, Eric A. Epping, and David J. Moser declare that they have no conflict of interest. Unrelated to this manuscript, Dr. Helen S. Mayberg has served as a consultant for and receives intellectual property licensing fees from St. Jude Medical Inc.

## Abbreviations

AVD: atherosclerotic vascular disease
df: degrees of freedom
FA: fractional anisotropy
MD: mean diffusivity
SCL-90-R: Symptom Checklist 90 – Revised
vAC: ventral anterior cingulate.

## References

[B1] Alexopoulos G. S. Meyers B. S. Young R. C. Campbell S. Silbersweig D. Charlson M. (1997). ‘Vascular depression’ hypothesis. *Arch. Gen. Psychiatry* 54 915–922. 10.1001/archpsyc.1997.01830220033006 9337771

[B2] Alpers B. J. Berry R. G. Paddison R. M. (1959). Anatomical studies of the Circle of Willis in normal brain. *AMA Arch. Neurol. Psychiatry* 81 409–418. 10.1001/archneurpsyc.1959.02340160007002 13636509

[B3] Assaf Y. Pasternak O. (2008). Diffusion tensor imaging (DTI)-based white matter mapping in brain research: a review. *J. Mol. Neurosci.* 34 51–61. 10.1007/s12031-007-0029-0 18157658

[B4] Bae J. N. MacFall J. R. Krishnan R. R. Payne M. E. Steffens D. C. Taylor W. D. (2006). Dorsolateral prefrontal cortex and anterior cingulate cortex white matter alterations in late-life depression. *Biol. Psychiatry* 60 1356–1363. 10.1016/j.biopsych.2006.03.052 16876144

[B5] Basser P. J. Pierpaoli C. (1996). Microstructural and physiological features of tissues elucidated by quantitative-diffusion-tensor MRI. *J. Magn. Reson. B* 111 209–219. 10.1006/jmrb.1996.0086 8661285

[B6] Beck A. T. Steer R. A. Garbin M. G. (1988). Psychometric properties of the Beck depression inventory – 25 years of evaluation. *Clin. Psychol. Rev.* 8 77–100. 10.1016/0272-7358(88)90050-5

[B7] Bijanki K. R. Arndt S. Magnotta V. A. Nopoulos P. Paradiso S. Matsui J. T. (2013a). Characterizing white matter health and organization in atherosclerotic vascular disease: a diffusion tensor imaging study. *Psychiatry Res.* 214 389–394. 10.1016/j.psychresns.2013.07.011 24144509 PMC4175449

[B8] Bijanki K. R. Stillman A. N. Arndt S. Magnotta V. A. Fiedorowicz J. G. Haynes W. G. (2013b). White matter fractional anisotropy is inversely related to anxious symptoms in older adults with atherosclerosis. *Int. J. Geriatr. Psychiatry* 28 1069–1076. 10.1002/gps.3930 23348834 PMC3690172

[B9] Bijanki K. R. Hodis B. Brumm M. C. Harlynn E. L. McCormick L. M. (2014). Hippocampal and left subcallosal anterior cingulate atrophy in psychotic depression. *PLoS ONE* 9:e110770 10.1371/journal.pone.0110770 PMC4206433 25338068

[B10] Catani M. ffytche D. H. (2005). The rises and falls of disconnection syndromes. *Brain* 128 2224–2239. 10.1093/brain/awh622 16141282

[B11] Cercignani M. (2011). “Strategies for patient-control comparison of diffusion MRI data,” in *Diffusion MRI: Theory, Methods, and Applications,* ed. Jones D. (New York, NY: Oxford University Press), 485–500.

[B12] Cheng P. Magnotta V. A. Wu D. Nopoulos P. Moser D. J. Paulsen J. (2006). Evaluation of the GTRACT diffusion tensor tractography algorithm: a validation and reliability study. *Neuroimage* 31 1075–1085. 10.1016/j.neuroimage.2006.01.028 16631385

[B13] Derogatis L. R. Melisaratos N. (1983). The brief symptom inventory – an introductory report. *Psychol. Med.* 13 595–605. 10.1017/S0033291700048017 6622612

[B14] Dougherty D. D. Weiss A. P. Cosgrove G. R. Alpert N. M. Cassem E. H. Nierenberg A. A. (2003). Cerebral metabolic correlates as potential predictors of response to anterior cingulotomy for treatment of major depression. *J. Neurosurg.* 99 1010–1017. 10.3171/jns.2003.99.6.1010 14705729

[B15] Drevets W. C. Price J. L. Simpson J. R. Todd R. D. Reich T. Vannier M. (1997). Subgenual prefrontal cortex abnormalities in mood disorders. *Nature* 386 824–827. 10.1038/386824a0 9126739

[B16] Firbank M. J. Teodorczuk A. van der Flier W. M. Gouw A. A. Wallin A. Erkinjuntti T. (2012). Relationship between progression of brain white matter changes and late-life depression: 3-year results from the LADIS study. *Br. J. Psychiatry* 201 40–45. 10.1192/bjp.bp.111.098897 22626634

[B17] Geschwind N. (1965). Disconnexion syndromes in animals and man. *Brain* 88 237–294. 10.1093/brain/88.2.237 5318481

[B18] Greicius M. D. Flores B. H. Menon V. Glover G. H. Solvason H. B. Kenna H. (2007). Resting-state functional connectivity in major depression: abnormally increased contributions from subgenual cingulate cortex and thalamus. *Biol. Psychiatry* 62 429–437. 10.1016/j.biopsych.2006.09.020 17210143 PMC2001244

[B19] Grool A. M. Gerritsen L. Zuithoff N. P. A. Mali W. P. van der Graaf Y. Geerlings M. I. (2013). Lacunar infarcts in deep white matter are associated with higher and more fluctuating depressive symptoms during three years follow-up. *Biol. Psychiatry* 73 169–176. 10.1016/j.biopsych.2012.08.024 23079234

[B20] Harris G. Andreasen N. C. Cizadlo T. Bailey J. M. Bockholt H. J. Magnotta V. A. (1999). Improving tissue classification in MRI: a three-dimensional multispectral discriminant analysis method with automated training class selection. *J. Comput. Assist. Tomogr.* 23 144–154. 10.1097/00004728-199901000-00030 10050826

[B21] Iwabuchi S. J. Häberling I. S. Badzakova-Trajkov G. Patston L. L. M. Waldie K. E. Tippett L. J. (2011). Regional differences in cerebral asymmetries of human cortical white matter. *Neuropsychologia* 49 3599–3604. 10.1016/j.neuropsychologia.2011.09.011 21939675

[B22] Johansen-Berg H. Gutman D. A. Behrens T. E. Matthews P. M. Rushworth M. F. Katz E. (2008). Anatomical connectivity of the subgenual cingulate region targeted with deep brain stimulation for treatment-resistant depression. *Cereb. Cortex* 18 1374–1383. 10.1093/cercor/bhm167 17928332 PMC7610815

[B23] Kennedy S. H. Konarski J. Z. Segal Z. V. Lau M. A. Bieling P. J. McIntyre R. S. (2007). Differences in brain glucose metabolism between responders to CBT and venlafaxine in a 16-week randomized controlled trial. *Am. J. Psychiatry* 164 778–788. 10.1176/appi.ajp.164.5.778 17475737

[B24] Khalaf A. Edelman K. Tudorascu D. Andreescu C. Reynolds C. F. Aizenstein H. (2015). White matter hyperintensity accumulation during treatment of late life depression. *Neuropsychopharmacology* 10.1038/npp.2015.158 [Epub ahead of print]. PMC4864637 26058663

[B25] Krishnan K. R. Hays J. C. Blazer D. G. (1997). MRI-defined vascular depression. *Am. J. Psychiatry* 154 497–501. 10.1176/ajp.154.4.497 9090336

[B26] Magnotta V. A. Harris G. Andreasen N. C. O’Leary D. A. Yuh W. T. C. Heckel D. (2002). Structural MR image processing using the BRAIN2 toolbox. *Comput. Med. Imaging Graph.* 26 251–264. 10.1016/s0895-6111(02)00011-3 12074920

[B27] Mayberg H. S. (2009). Targeted electrode-based modulation of neural circuits for depression. *J. Clin. Invest.* 119 717–725. 10.1172/jci38454 19339763 PMC2662569

[B28] Mayberg H. S. Liotti M. Brannan S. K. McGinnis S. Mahurin R. K. Jerabek P. A. (1999). Reciprocal limbic-cortical function and negative mood: converging PET findings in depression and normal sadness. *Am. J. Psychiatry* 156 675–682. 10.1176/ajp.156.5.675 10327898

[B29] Mayberg H. S. Lozano A. M. Voon V. McNeely H. E. Seminowicz D. Hamani C. (2005). Deep brain stimulation for treatment-resistant depression. *Neuron* 45 651–660. 10.1016/j.neuron.2005.02.014 15748841

[B30] Mayberg H. S. Silva J. A. Brannan S. K. Tekell J. L. Mahurin R. K. McGinnis S. (2002). The functional neuroanatomy of the placebo effect. *Am. J. Psychiatry* 159 728–737. 10.1176/appi.ajp.159.5.728 11986125

[B31] McCormick L. M. Ziebell S. Nopoulos P. Cassell M. Andreasen N. C. Brumm M. (2006). Anterior cingulate cortex: an MRI-based parcellation method. *Neuroimage* 32 1167–1175. 10.1016/j.neuroimage.2006.04.227 16859929

[B32] Meng X. L. Rosenthal R. Rubin D. B. (1992). Comparing correlated correlation-coefficients. *Psychol. Bull.* 111 172–175. 10.1037/0033-2909.111.1.172

[B33] Mitchell R. N. Schoen F. J. (2010). “Blood vessels: atherosclerosis,” in *Robbins and Cotran Pathologic Basis of Disease*, 8th Edn, eds Kumar V. K. Abbas A. K. Fausto N. Aster J. C. (Philadelphia, PA: Saunders Elsevier), 496–507. 10.1016/b978-1-4377-0792-2.50016-x

[B34] Mori S. Van Zijl P. (2007). Human white matter atlas. *Am. J. Psychiatry* 164 1005 10.1176/ajp.2007.164.7.1005 17606649

[B35] Moser D. J. Robinson R. G. Hynes S. M. Reese R. L. Arndt S. Paulsen J. S. (2007). Neuropsychological performance is associated with vascular function in patients with atherosclerotic vascular disease. *Arterioscler. Thromb. Vasc. Biol.* 27 141–146. 10.1161/01.atv.0000250973.93401.2d 17068287

[B36] Musselman D. Evans D. Nemeroff C. (1998). The relationship of depression to cardiovascular disease - epidemiology, biology, and treatment. *Arch. Gen. Psychiatry* 55 580–592. 10.1001/archpsyc.55.7.580 9672048

[B37] Nobler M. S. Oquendo M. A. Kegeles L. S. Malone K. M. Campbell C. Sackeim H. A. (2001). Decreased regional brain metabolism after ECT. *Am. J. Psychiatry* 158 305–308. 10.1176/appi.ajp.158.2.305 11156816

[B38] Ongur D. Drevets W. C. Price J. L. (1998). Glial reduction in the subgenual prefrontal cortex in mood disorders. *Proc. Natl. Acad. Sci. U.S.A.* 95 13290–13295. 10.1073/pnas.95.22.13290 9789081 PMC23786

[B39] Pantoni L. Garcia J. H. Gutierrez J. A. (1996). Cerebral white matter is highly vulnerable to ischemia. *Stroke* 27 1641–1646. 10.1161/01.STR.27.9.1641 8784142

[B40] Pfefferbaum A. Adalsteinsson E. Sullivan E. V. (2005). Frontal circuitry degradation marks healthy adult aging: evidence from diffusion tensor imaging. *Neuroimage* 26 891–899. 10.1016/j.neuroimage.2005.02.034 15955499

[B41] Rajkowska G. Miguel-Hidalgo J. J. (2007). Gliogenesis and glial pathology in depression. *CNS Neurol. Disord. Drug Targets* 6 219–233. 10.2174/187152707780619326 17511618 PMC2918806

[B42] Riva Posse P. Choi K. S. Holtzheimer P. E. McIntyre C. C. Gross R. E. Chaturvedi A. (2014). Defining critical white matter pathways mediating successful subcallosal cingulate deep brain stimulation for treatment-resistant depression. *Biol. Psychiatry* 76 963–969. 10.1016/j.biopsych.2014.03.029 24832866 PMC4487804

[B43] Robinson R. G. Price T. R. (1982). Post-stroke depressive disorders: a follow-up study of 103 outpatients. *Stroke* 13 635–641. 10.1161/01.STR.13.5.635 7123596

[B44] Rudebeck P. H. Putnam P. T. Daniels T. E. Yang T. Mitz A. R. Rhodes S. E. V. (2014). A role for primate subgenual cingulate cortex in sustaining autonomic arousal. *Proc. Natl. Acad. Sci. U.S.A.* 111 5391–5396. 10.1073/pnas.1317695111 24706828 PMC3986148

[B45] Shimony J. S. Sheline Y. I. D’Angelo G. Epstein A. A. Benzinger T. L. Mintun M. A. (2009). Diffuse microstructural abnormalities of normal-appearing white matter in late-life depression: a diffusion tensor imaging study. *Biol. Psychiatry* 66 245–252. 10.1016/j.biopsych.2009.02.032 19375071 PMC2804471

[B46] Steer R. A. Clark D. A. Ranieri W. F. (1994). Symptom dimensions of the SCL-90-R: a test of the tripartite model of anxiety and depression. *J. Pers. Assess.* 62 525–536. 10.1207/s15327752jpa6203_12 8027915

[B47] Taylor W. D. Aizenstein H. J. Alexopoulos G. S. (2013). The vascular depression hypothesis: mechanisms linking vascular disease with depression. *Mol. Psychiatry* 18 963–974. 10.1038/mp.2013.20 23439482 PMC3674224

[B48] Taylor W. D. MacFall J. R. Payne M. E. McQuoid D. R. Provenzale J. M. Steffens D. C. (2004). Late-life depression and microstructural abnormalities in dorsolateral prefrontal cortex white matter. *Am. J. Psychiatry* 161 1293–1296. 10.1176/appi.ajp.161.7.1293 15229065

[B49] Taylor W. D. MacFall J. R. Provenzale J. M. Payne M. E. McQuoid D. R. Steffens D. C. (2003). Serial MR imaging of volumes of hyperintense white matter lesions in elderly patients: correlation with vascular risk factors. *Am. J. Roentgenol.* 181 571–576. 10.2214/ajr.181.2.1810571 12876050

[B50] Zald D. H. Mattson D. L. Pardo J. V. (2002). Brain activity in ventromedial prefrontal cortex correlates with individual differences in negative affect. *Proc. Natl. Acad. Sci. U.S.A.* 99 2450–2454. 10.1073/pnas.042457199 11842195 PMC122385

