# Supplementary figures and images for: Depressive symptoms related to low fractional anisotropy of white matter underlying the right ventral anterior cingulate in older adults with atherosclerotic vascular disease
Source: Front Hum Neurosci. 2015 Jul 15;9:408. doi: 10.3389/fnhum.2015.00408 (PMC4502350; doi:10.3389/fnhum.2015.00408)

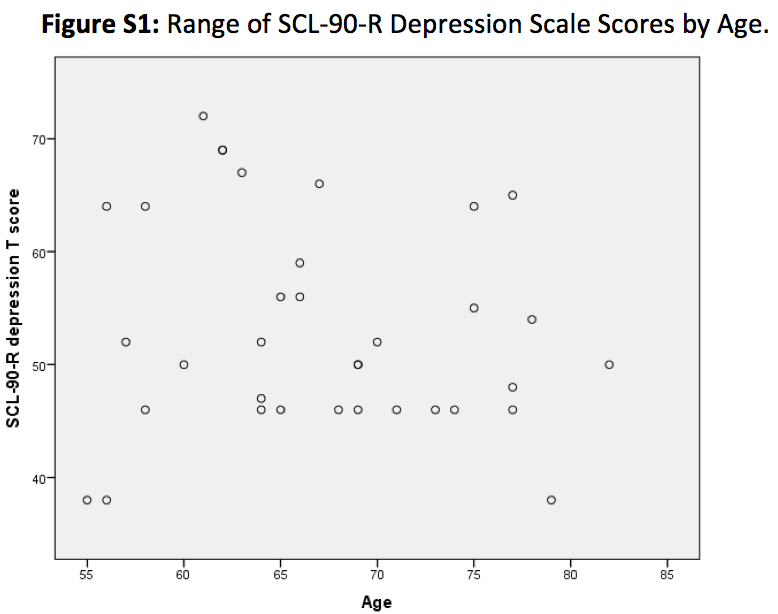

Supplement: Supplementary file 1 [file Image_1.TIFF]
